# Self-empowerment of life through RNA networks, cells and viruses

**DOI:** 10.12688/f1000research.130300.1

**Published:** 2023-02-06

**Authors:** Luis Villarreal, Guenther Witzany

**Affiliations:** 1 Center for Virus Research, University of California, Irvine, California, USA; 2 Telos - Philosophische Praxis, Buermoos, Salzburg, 5111, Austria

**Keywords:** RNA stem-loop groups, social behavior, biocommunication, gangs of life, memory, learning, excess productivity

## Abstract

Our understanding of the key players in evolution and of the development of all organisms in all domains of life has been aided by current knowledge about RNA stem-loop groups, their proposed interaction motifs in an early RNA world and their regulative roles in all steps and substeps of nearly all cellular processes, such as replication, transcription, translation, repair, immunity and epigenetic marking. Cooperative evolution was enabled by promiscuous interactions between single-stranded regions in the loops of naturally forming stem-loop structures in RNAs. It was also shown that cooperative RNA stem-loops outcompete selfish ones and provide foundational self-constructive groups (ribosome, editosome, spliceosome, *etc*.). Self-empowerment from abiotic matter to biological behavior does not just occur at the beginning of biological evolution; it is also essential for all levels of socially interacting RNAs, cells and viruses.

## 1. Introduction

In the 20 ^th^ century, when biology was a subdiscipline of physics and chemistry, it was common to choose explanatory models that helped us understand evolutionary processes in terms of gradual steps from abiotic physical reactions to biological variation and selection processes ( Eigen, 1971; Schuster, 2011). With the rise of quasispecies theory and research into the behavior of RNA viruses, we now know that RNA group interaction motifs “resemble, in many ways, social behavior” (see Eigen, 1971, p. 505). We can use a more up-to-date description that integrates an abundance of signal-mediated interactions into a more coherent picture. These interactions range from social RNA networks and cell–cell communication, to communication of viruses ( Villarreal & Witzany, 2010, 2013a, 2015; Witzany, 2015). This picture contains features that are not exclusively the domain of mathematics, physics and chemistry but also come under empirical social sciences that help us better understand various behavioral motifs in group interactions ( Díaz-Muñoz, Sanjuán, & West, 2017; Witzany, 2016a).

## 2. At the evolutionary beginning of biotic behavior

Successful concepts about prebiotic evolution and its various implications have been reviewed elsewhere and will not be repeated here ( Oparin, 1965; Oparin & Gladilin, 1980; Mathis *et al.,* 2017; Vlassov *et al.,* 2004; Lehman, 2013). We are more interested in the real beginnings of biological evolution. Before the start of biotic processes, we can identify a kind of self-organization of matter without biotic features: the snap-back of single-stranded RNA molecules. This mechanism is found when a few single-stranded RNA molecules fold back within their own identity, to form a double-stranded RNA stem and a single-stranded RNA loop at the folding angle ( Manrubia & Briones, 2007; Mattick & Amaral, 2022; Stich, Briones, & Manrubia, 2007; Levin, Gandon & West, 2020). This results in the basic structure of all subsequent following RNA stem-loop group interaction motifs ( Moore, 1999; Müller *et al.*, 2012; Petkovic & Müller, 2013). The double-stranded RNA stem is not prone to binding based on the complementarity of RNA syntax. In contrast, the single-stranded RNA loop is somewhat prone to binding as it complementarily binds to other single-stranded RNAs. The snap-back functions exclusively according to the laws of physics and chemistry. No biological selection occurs at this stage. It clearly represents self-organization of matter.

**Figure 1.**
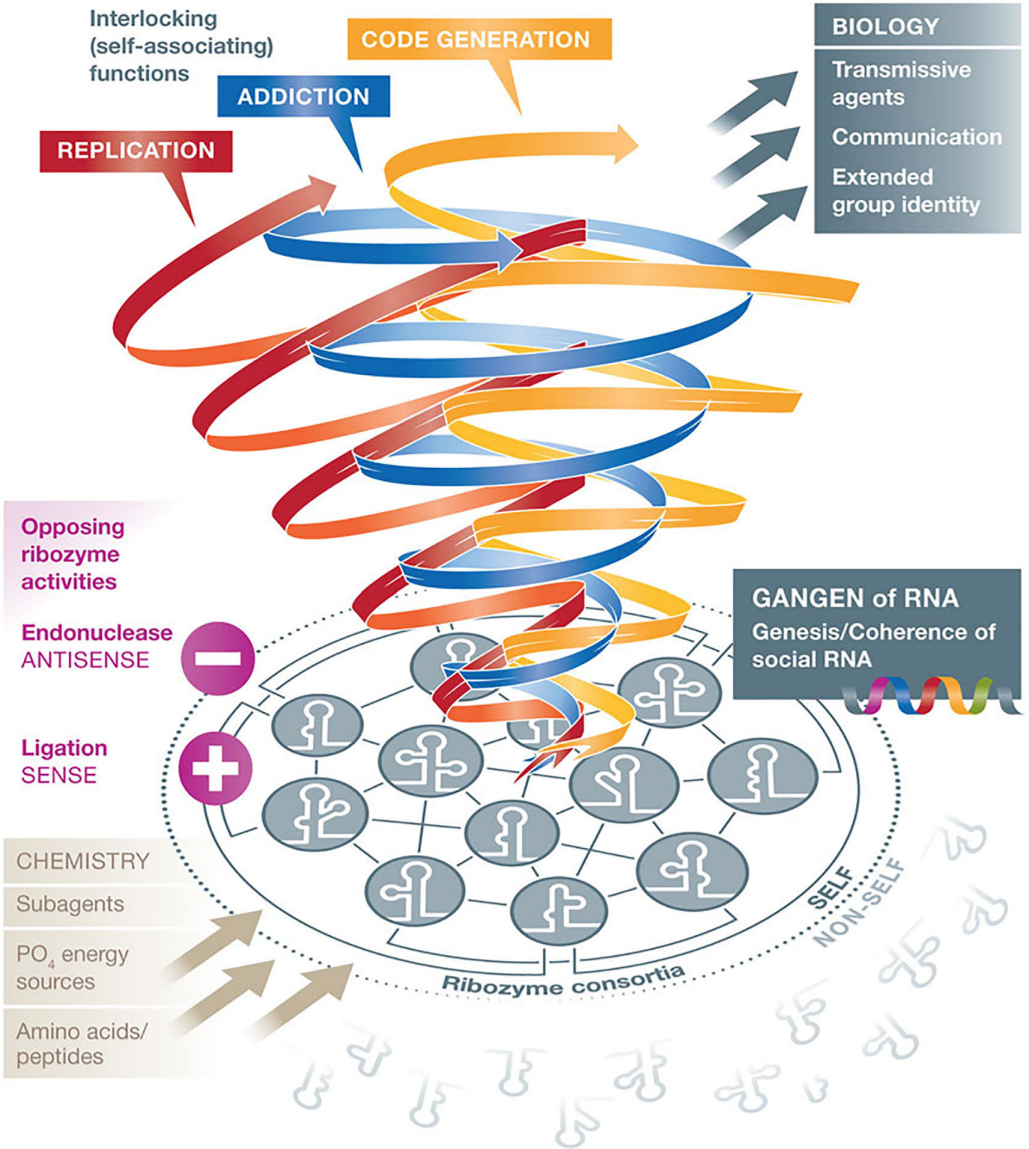
The RNA “gangen” hypothesis: RNA stem-loop groups assemble like social gangs. According to Villarreal, group identity and cooperativity of an RNA collective require opposing functions which are essential for the genesis of life (social behavior of agents). Interestingly, at the very beginning of the RNA world, ribozyme consortia with group identities initiated the differentiation competence of “self” from “non-self”, like “gangs”, according to dynamically varying contextual requirements. Figure 1 assembles all key topics: abiotic–biotic interactions, consortial life and identity building, inclusion–preclusion, opposing ribozyme activities (endonuclease–ligase), basics of cell biology and code emergence out of interactions (from Villarreal, 2015; with permission by the New York Academy of Sciences).

The RNA stem-loops have several distinct parts/subunits: stems consisting of base-paired nucleotides and loops/bulges/junctions consisting of unpaired regions limited by stems. It is important to note that any RNA is part of such stem-loops. Biological selection processes emerge in the presence of a certain density of RNA stem-loops. Then RNA stem-loops cooperate and compete, which are behavioral motifs that are completely absent in abiotic matter (see Figure 1). This means that biological selection is clearly a result of social interactions and starts not with the first living cell or with LUCA (last universal common ancestor), but earlier in the RNA world. An unexpected finding is that cooperative RNA stem-loops outcompete selfish ones ( Smit, Yarus & Knight, 2006; Hayden & Lehman, 2006; Higgs & Lehman, 2014; Vaidya *et al.,* 2012; Vaidya, Walker & Lehman, 2013; Witzany, 2016b).

The abundance of RNA agents that form cooperative groups seem to have their first evolutionary revolution in forming the tRNA clover loop with three stem-loop arms, which is a forerunner of the more complex assembly of ribosomal subunits ( Wegrzyn & Wegrzyn, 2008; Kanai, 2015). The clover leaf consists of an acceptor stem, D-arm, the anticodon arm, the variable loop, and the T-arm, also known as the TΨC arm. Each arm consists of a double-stranded stem and a single-stranded loop ( Root-Bernstein, Kim, Sanjay, & Burton, 2016; Kim *et al.,* 2017; Sun & Caetano-Anolles, 2007; Di Giulio, 2012). tRNAs appear to represent the oldest consortia of RNA stem-loop groups and are derived from two different parts that originally emerged for purposes other than the translation of mRNAs into polyaminoacid sequences ( Caetano-Anolles & Sun, 2014; Root-Bernstein & Root-Bernstein, 2019; Demongeot & Seligman, 2019; Dantas, José, & de Farias, 2021). As tRNAs are one of the most prominent RNA groups with a long history, this structure has been conserved for all organisms thanks to the evolutionary, successful and therefore beneficial selection ( Schmidt & Matera 2020). It is also interesting to note that the pre-tRNAs contain introns that must be spliced out before a final mature tRNA can start translation processes ( Hayne, Lewis & Stanley, 2022).

### 2.1 Ligated RNA stem-loop groups: the ribosome

The self-ligation of different parts of RNA stem-loop groups occurs at single-stranded RNA parts ( Gwiazda *et al*., 2012). Here, it is important to note that circular RNAs are protected against endonuclease degradations and therefore represent an evolutionary benefit in this early stage of the RNA world ( Lasda & Parker, 2014; Müller, 2015; Lee & Koonin, 2022; Rivera-Madrinan, Di Iorio, & Higgs, 2022). With the emergence of the RNA polymerase ribozyme, functional RNA molecules, including the polymerase itself, could be copied ( Attwater, Wochner, & Holliger, 2013; Tjhung, Shokhirev, Horning, & Joyce, 2020). Early in the RNA world an important player emerged, RNAse H, which may have evolved from ribozymes, related to viroids and forming ribosomes. Even today, RNAse H mediates a variety of functions in all domains of life, including the virosphere ( Moelling, Broecker, Russo, & Sunagawa, 2017). RNA stem-loop groups thus lay the foundations for complex essential ‘-some’ activities, starting with ribosomes.

With the emergence of ribosomes, the next biological revolution took place: protein translation. The RNA polymerase has a close interaction with the ribosome, which helps to maintain genome integrity and conserves energy during transcription and translation ( McGary & Nudler, 2013). The rather precise translation into proteins paved the way to a wide range of cellular domains ( Gordon, 1995; Davidovich, Belousoff, Bashan, & Yonath, 2009; Bernhardt, & Tate, 2010; Sun & Caetano-Anollés, 2008). Interestingly, all known cells share this ribosomal feature, but no virus contains it, although RNA stem-loop groups in the long evolutionary process not only assembled but also genetically fixed some ribosomal subunits ( Mizuno *et al*., 2019; Bowman *et al*., 2020). Furthermore, the ribosome is a ribozyme that is at the core of the translation into proteins which do the most work in cell biology. It enables the tRNA to build a series of polypeptides of amino acids, which are the basics for all functions of cellular organisms. In addition, the formation of a tri-peptide at the tRNA is a concerted action at the center of this ribosome ribozyme. Meanwhile, the evolutionary history of the ribosomal subunits has been extensively investigated ( Randau & Söll, 2008; Harish & Caetano-Anolles, 2012; Petrov *et al*., 2015). Each of these big consortia of single RNA stem-loops can be studied according to context and history, which means that the various very old parts and the younger parts of the ribosomal subunits can be analyzed ( Ariza-Mateos *et al*., 2019). The question remains as to how and why these two different consortia are unified and conserved in any cellular life.

### 2.2 Pre-mRNA processing

The messenger RNA derives from a pre-stage as the primary transcript out of the DNA of a gene performed and produced by an RNA polymerase. Without polymerases, no single DNA strand can be processed into an mRNA. Within the pre-mRNA primary transcript, we can find the protein-coding sequences (non-repetitive) and the introns (repetitive) that separate the final mRNA, ready to be translated into the polypeptide strain. The evolutionary split of RNA sequences into repetitive and non-repetitive nucleotide syntax had far-reaching consequences for the evolution of protein-coding complexity and regulation ( Shapiro & von Sternberg, 2005; Jurka *et al*., 2007; Witzany, 2017a).

Various highly coordinated processes are outlined by different RNA networks, such as RNA editing (editosome) and alternative splicing (spliceosome). Both the editosome and the spliceosome represent RNA stem-loop groups that assembled through ligation procedures during their long evolutionary history ( Gott, 2003; Matlin & Moore, 2007; Alfonzo, 2008; Hesselberth, 2013). This history is illustrated by the variety of, for example, six subunits of the final spliceosome (a group of nuclear RNAs). Spliceosomal actions take place after the RNA editing by the editosome. Splicing and editing are heavily interconnected and provide variable meanings of the identical DNA sequence. For translation into cellular organisms, a line-up of all the exons must be produced in which the introns are cut out. The sites where splicing of the introns occurs must be exactly identified ( Matera & Wang, 2014; Izquierdo & Valcárcel, 2006). Interestingly, small nuclear RNAs (snRNA) base-pair with short sequences in pre-mRNAs to mark the sequences to be spliced out. A total of 200 small nucleolar RNAs are known to act as guides ( Zhang *et al*., 2019). They are encoded in introns and transcribed by RNA polymerase II. In some organisms, these introns are conserved more than the exons. Following the splicing processes, the remaining RNAs are recycled for other catalytic processes.

### 2.3 The current RNA sphere

RNA stem-loop groups represent an unmanageable quantity of sophisticated regulatory networks ( Mattick & Amaral, 2022). These groups are crucial in the following functions:

• DNA replication with important functions of centromeres and telomeres in genome maintenance
• RNA guidance of chromosome structure
• Regulation of transcriptional and post-transcriptional modifications by spliceosomes and editosomes according to the requirements of the context
• Regulatory pathways and coordination in all steps of the translation into proteins
• Epigenetic marking and short-term and long-term memory formation and its (re)modification
• DNA repair organization and coordination in all detailed steps and substeps
• Immunity organization and coordination in all steps and substeps by genome plasticity, V (ariable) D (iversity) J (oining) plasticity in adaptive immune response
• Genetic identity of organisms which initiates motifs of self-interaction or non-self-interaction
• Genetic content composition of host organisms by genetic parasites (viruses and defectives such as transposons and retroposons)
• Intron/exon genome fragmentation as a benefit in immune functions (CRISPR/Cas) as well as in genome modularity and complexity.

The active roles played by RNA stem-loop groups ensure all life processes currently known and start with the transcription process out of the relatively stable DNA storage medium. After transcription, an abundance of RNA stem-loop variants are available and interact in well-coordinated actions. The folding loop remains as a single-stranded RNA sequence. Various motifs have been identified, yet all of them share a common function: they stabilize RNA tertiary formation. Such motifs include:

• pseudoknots, kissing loops, A-minor motifs, A-platforms, kink-turns, S-turns, tetraloops and their receptors, and a variety of non-canonical base-pairs and base-triples
• ribosomal frameshift as a natural technique to process alternative translation of an mRNA sequence by changing the open-reading frame
• bypassing translation
• competing endogenous RNAs.

All these highly coordinated and interconnected motifs of RNA stem-loop groups may alter the meaning of the information stored in the DNA according to the environmental and/or circumstantial requirements of an organism, which means that the information is context dependent ( Witzany, 2020a).

Group identity and membership of single RNA stem-loops in ensembles of formerly integrated groups seem to be useful in (re)building new RNA stem-loop groups. We must not forget the essential roles that RNA minorities such as former degraded RNAs play in small RNA stem-loop groups, or even single stem-loops ready to be reused in (re)building current functional RNA groups.

### 2.4 Code and code editors

The genetic code of DNA may represent the result of the effective escape strategy of RNA-networks: out of a competitive RNA world and into a new sequence space to better conserve successfully selected genetic identities. The reverse transciptases and related RNA networks produce complementary strands of RNA to DNA, which means it’s the crucial escape technique. As RNA polymerases they copy a single stranded RNA sequence into a single stranded DNA sequence. The reverse transcriptase is very old and a key driver of genomic plasticity and seems to stem from a retroviral origin ( Hu & Temin, 1990; Brosius & Tiedge, 1995; Aziz, Breitbart & Edwards, 2010). In this perspective the DNA world could have startet with a retroprocessing competence, later on transferred to cellular organisms by retroviruses, retrotransposons and related genetic parasites.

This means DNA can also be investigated as fixed (memorized) evolutionary results arising from RNA stem-loop group competition and cooperation. In this perspective, DNA-based information represents a natural code, and RNA stem-loop groups represent the code editors. This makes sense because if DNA is really a natural code, we must not forget that no natural code codes itself, just as no natural language speaks itself ( Witzany, 2014a; Nelson & Breaker, 2017). In all empirical observations to date, there are competent agents that edit, use, reuse or create natural code sequences or natural language-based sequence constructions ( Witzany, 2015) Accordingly, the epigenetic markings of such DNA sequences represent how DNA-based information is programmed, to produce proteins and to produce RNAs that regulate the production of proteins according to the contextual requirements. This means that the epigenetic programming determines the meaning (function) of the sequence content to be realized ( Nowacki, Shetty & Landweber, 2011).

### 2.5 Context-dependent membership roles

In contrast to mathematical modelling, socially interacting RNA stem-loop groups may help us better understand group membership and interactional motifs to establish relevant genetic identities ( Villarreal, 2009a; Villarreal & Witzany, 2021) RNA sociology:

• may identify the key players that edit (modify and determine) the genetic codes of host genomes as consortia of RNA agents and virus-like genetic parasites that drive evolutionary novelty;
• investigates RNA group behavioral motifs and their role in regulating replication, gene expression, recombination, immune functions and generation of new nucleotide sequences;
• explains the emergence of new sequence space not as a result of selection arising from error-replication events, but as a result of competent generation of nucleotide sequences and genome editing;
• focuses on social behavior of RNA agents with their emerging properties, such as communicative interactions represented by cooperation and competition ( Villarreal & Witzany, 2013a; Witzany, 2014b).

The natural code and its users and their communicative interactions can be investigated as social phenomena which cannot be fully understood by mathematical modelling (see chapter 3) and physical/chemical investigations alone, as these focus on statistical mechanics which differ from biotic behavior ( Witzany, 2020b; Villarreal, 2015).

## 3. The outdated 20 ^th^ century narrative

In groundbreaking articles Manfred Eigen and Peter Schuster led foundation to the quasi-species concept ( Eigen, 1971). In detail they investigated how biological macromolecules, *i.e.* RNA stem-loop groups interact and self reproduce. They were convinced that the whole object of investigation principally can be fully described in terms of physics, which means, can be computed and depicted in mathematical equations ( Eigen & Schuster, 1978). For the authors there was no doubt that biology remains a subdiscipline of physics, and also darwinian principles can be fully understood by formalizable equations. Therefore it was logical to use the formal analogy of quasispecies dynamics and statistical mechanics ( Domingo & Schuster, 2016). Biological selection then can be explained as condensation (localisation) of sequence distribution in a limited area in a formal sequence space. Evolution theory would then be based on biochemical kinetics ( Biebricher, Eigen & Gardiner Jr., 1985).

Eigen subsumed generation of information under a dynamic theory of matter ( Eigen & Winkler, 1983). Because reproduction processes in a self-reproducing matter depend on limited energy resources it would be natural that such self-reproducing processes underly a certain error rate which is the basis for variations of a master copy. The evolution of living systems with quasispecies dynamics and proteins depends on an unequivocal “code-system” and a relational protein system which evolves *via* “hypercycles”. This leads to the Eigen-Schuster equations in which evolutionary results are coherent with natural laws of physics ( Eigen, 2013). Errors in this system of reproduction are the result of an instability within the system which can be sufficiently explained by irreversibe thermodynamic processes ( Eigen, 1971).

### 3.1 Life as a self-reproducing machine?

Eigen refers the explanatory model of the self-reproducing machine to a reality in which these automatons meet the requirements of Darwin’s theory of biological evolution ( Eigen & Winkler,1983). He is convinced that without no doubt John von Neumann’s concept of a self-reproducing machine represents a mathematically exact refinement of Alan Turing’s idea of a self-reproducing machine ( Eigen, 2013). Some decades later Sydney Brenner argues again that cells and living organisms are good examples of Turing and von Neumann machines ( Brenner, 2012). But living nature cannot be properly accommodated within such a theoretical framework ( Witzany & Baluska, 2012a). Although proposed for more than 80 years not even one self-reproducing machine has been constructed or seen until today. Empirical data contradict this model of explanation.

Eigen’s adaptation of this machine thinking to living agents was a misconception because the language that codes machine programs is not compatible with that of the genetic code. Languages controlling Turing and von Neumann machines are based on formal algorithms, in which syntax determines meaning independently of context ( Witzany, 1995). But as epigenetics demonstrated, gene expression essentially depends on environmental context and cannot be similarly treated as a formal language. A formal language syntax which can be expressed by mathematical equations transports unequivocal meanings. In contrast natural languages and codes represent both, a superficial grammar and a hidden deep grammar with varying context dependent meanings ( *e.g.*, “The Shooting of the Hunters.”). Not the superficial DNA sequence syntax determines the meaning (function) but pragmatic context of real life in which organisms are involved. This is a crucial difference in explaining genetic information. In the explanatory models of the 20 ^th^ century, the superficial DNA syntax was thought to represent the final information not its pragmatic use with its varying context dependent meanings. In contrast to 20 ^th^ century narratives which tried to identify meaning out of superficial sign sequence syntax, we now know that it is the context in which sign sequences are used that determines the meaning, not the syntax. This was completely ignored by linguist Chomsky and chemist Eigen ( Chomsky, 1965; Witzany, 1995). As a consequence, the mathematical equations of the quasispecies concept favoring a mutant spectra out of a fittest master type must be revised accordingly ( Villarreal & Witzany, 2013b). It is correct that RNA group interaction motifs “resemble, in many ways, social behavior” as proposed by Eigen. But social behavior of living agents does not represent interactions of self-reproducing machines that are programmed by algorithm-based procedures.

## 4. Cellular organisms communicate in any coordination

After this clarification of how RNA-groups interact, we now look at cellular life which represents the planetary ecosphere for RNA mediated interactions and regulations. The explosion of knowledge about the early and the current RNA world has not led to a better model explaining how cellular life started out of this RNA world ( Manrubia *et al.*, 2021) What we know now is that the essential processes in cellular organisms are regulated by RNA-mediated processes, whether these be the various steps of transcription and translation, DNA repair, immunity, replication and epigenetic markings. In particular, the epigenetic programming of cellular developmental stages is a crucial step in understanding cellular life and designates the epicenter of genetic information ( Mattick & Amaral, 2022).

With the evolution of cellular organisms, a new stage in the evolution of life was reached. Henceforth, stable storage of DNA genetic information was established not only as a blueprint for cellular reproduction processes, but also as a life habitat for an abundance of invading genetic parasites, whether at a persistent stage or a lytic stage ( Brookfield, 2005; Le Rouzic, Dupas & Capy, 2007; Vennera, Feschotte & Biemonta, 2009). With the start of cellular life, a completely new dimension originated, that of cellular populations that occupy ecological niches to metabolize and reproduce on this planet. The highly coordinated stages of all essential processes in the cell are stored in the DNA of each cell and are processed according to the different epigenetic markings. In addition, a DNA proofreading mechanism started, which had been absent in the early quasispecies era of RNA viruses and related genetic parasites.

Cellular organisms actively compete for environmental resources. They assess their surroundings, estimate how much energy they need for particular goals and then implement the optimum variant. They take measures to control certain environmental resources. They perceive themselves and can distinguish between “self” and “non-self”. Current empirical data on all domains of life indicate that unicellular organisms such as bacteria, archaea, giant viruses and protozoa, as well as multicellular organisms such as animals, fungi and plants, coordinate and organize their essential life functions through signaling processes ( Witzany, 2010, 2011a, 2012, 2014c, 2017b, 2020c; Witzany & Baluska, 2012b; Witzany & Nowacki, 2016).

Signaling allows for real-life coordination and organization. It is a communicative interaction in which species-specific behavioral patterns and sign repertoires are used according to three levels of rules (not laws) that govern the following: the combination of signs (syntax), the coherence between sign and content (semantics) and, most importantly, the use of signs according to a specific context (pragmatics). Rule-following in communicative interactions is essentially a social event, because a single agent cannot follow a rule only once, as proved by Ludwig Wittgenstein ( Wittgenstein, 1953). The use of signs in communicative interactions occurs by social sharing of a common set of signs and a commonly used set of rules.

**Figure 2.**
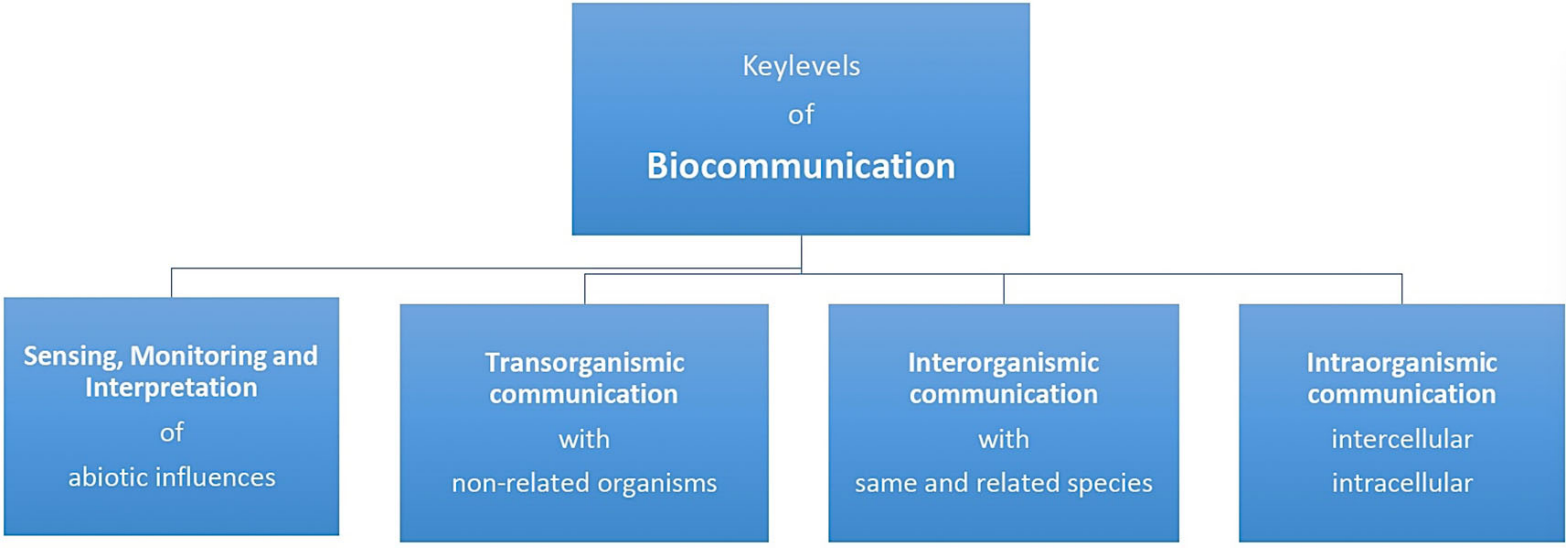
The biocommunication approach identified four levels of rule-governed and sign-mediated interactions in which cellular organisms are involved throughout their lives (from Witzany, 2010).

Cells, tissues, organs and organisms communicate at four key levels that are essential for all cell-based life forms (see Figure 2) ( Witzany, 2016a, 2019).

There is no doubt that all atoms and molecules on earth underlie the second law of thermodynamics. The generation of new sequence structures in natural codes, new behavior of organisms, new organs and tissues also underlie these laws. But this does not explain which communicative interactions between RNA networks, viruses and cells lead to new cells, tissues, organs and organisms, because natural laws do not change and adapt but remain fixed ( Salmena *et al*., 2011; Ariza-Mateos & Gomez, 2017; Villarreal & Witzany, 2019). In contrast, communicative rules in generation and combination of signs may change and adapt, which means that biotic innovations may be exapted and coopted for purposes which are different from those which previously emerged ( Frenkel-Pinter *et al*., 2022; Witzany, 2018) The main characteristic of sign-mediated interactions, in contrast to interactions not mediated by signs, is that the proponents may change the rules of sign use for purposes of adaptation, *e.g.* dialects in bacteria or even in bee languages, and generate really new sign sequences with really new content ( Ben Jacob *et al*., 2004; Schauder & Bassler, 2001; Witzany, 2014d; Baluska & Witzany, 2014). This represents a key feature in the self-empowerment of life.

Self-empowerment is the essence of biotic communication because it liberates socially interacting agents from the strictly dominating natural laws on abiotic planets and transforms these agents into a sphere of innovation, creativity and productivity to adapt to changing contexts. It is the inherent result of communicating agents, because they can generate really new results by the social use of natural codes, instead of following mechanistically from former conditions ( Witzany, 2019). Living agents use signs in natural communication processes, whether as indices, icons or symbols ( Atkin, 2010). They combine signs resulting in sign sequences and therefore increase information content and complexity. The contextual sign use by competent agents in populations constitutes meaning in all known natural languages or codes ( Prinz, 2022). And sign use in communication processes as well as the emergence of meaning is basically a social event. As previously mentioned, it is not possible that only a single agent invents signs or follows rules.

Signs in biotic communication processes within and between cells may be chemical molecules, electric and tactile signals, or also, as in higher animals, visual and auditive signals. More recently, it was found that cell–cell communication can also be mediated by small RNAs ( Lefebvre & Lécuyer, 2017, Buck, 2022) Additionally, abiotic influences, such as gravity, light, temperature, air movement, etc., also serve as signals to organisms which can be sensed, memorized and evaluated and may modify behavioral patterns.

### 4.1 Epigenetic memory and the emergence of learning processes

Genetic engineering in the 20 ^th^ century was dominated by the conviction that DNA sequences can be deleted, modified and inserted like molecular bricks to change the capabilities of certain plants, animals and other organisms. This conviction was supported by the one-gene–one-protein narrative, the perspective that non-coding RNAs represented “junk” and the central dogma of molecular biology, that means genetic information flows from DNA *via* RNA to protein, never reverse. This changed dramatically with the rise of epigenetics. The regulatory system that functions in the development, morphology, cell fate and identity, aging, physiology, genetic instructions, immunity, memory/learning, and physical and mental disease depends on epigenetic marks, DNA methylation and histone modifications ( Jablonka & Lamb, 2002; Cabej, 2018). Genetic sequences of all organisms in all domains of life can be marked according to their environmental and social experiences. This means a hidden layer of gene-regulating non-coding RNAs in organisms, which may also be inherited ( Jablonka & Lamb, 1992; Mattick, 2003; Shapiro, 2009).

Biotic memorizing is beneficial in helping organisms find better ways of adaptation and to circumvent mechanistic reproduction of “always the same” ( Skinner, 2014; Moore, 2016). This forms the basis for learning processes ( Zovkic, Guzman-Karlsson & Sweatt, 2013). Learning processes are a teleonomic key technique because they help organisms to react to experiences better than in previous, similar experiences ( Crisp *et al*., 2016; Landires & Núnez-Samudio, 2019; Jablonka, 2013). In the RNA-determined process on the genetic level, we may assume that the purposes of the assembled RNA group processes are directed not toward a singular goal but toward a competence, *i.e.*, a new capability to generate and test innovative tools of adaptation ( Marshall & Bredy, 2016; Mattick, 2010; Frías-Lasserre & Villagra, 2017; Stajic & Jansen, 2021). This new capability enables organisms to better adapt to environmental circumstances, with various behavioral patterns such as escape, invent, recombine, exapt, coopt, suppress, displace, compete and cooperate and their transgenerational inheritance ( Jablonka, 2013; Broecker, 2021).

Living organisms can adapt to environmental, ecological and random circumstances. This does not mean reproducing the inherited behavioral realms when reacting, but changing this behavior to better fit into a changing “umwelt” ( von Uexkuell, 1987) Organisms not only sense and monitor their experiences but even evaluate and compare present experiences with similar ones in the past on the epigenetic level ( Shapiro, 2014; McGowan & Roth, 2015). The result is a kind of evaluating comparison (interpretation) of sensory data according to various parameters ( Nowacki, Shetty & Landweber, 2011; Casadesús & D’Ari, 2002). If the resulting behavior is beneficial in contrast to that of those population members that do not integrate this interpretation, we may term this a successful learning process ( Miller & Sweatt, 2007). Learning means sensation, interpretation and adapted behavior, memory and monitoring against stored background information. Learning is a beneficial behavioral motif and outcompetes behavior without learning. Memory is contextual information storage, and learning is the transfer of comparative memory into everyday life ( Miller, Campbell & Sweatt, 2008). Without memory, learning is difficult because the comparative data in the background storage are not available. Memory is a pre-condition for learning. With memory and learning, living organisms have an important tool for survival.

Such memory/learning skills can be found in organisms ranging from viruses and akaryotes to unicellular eukaryotes, fungi, animals and plants, although it was only noted as a capability of higher animals until recently ( Csaba, 2017; Baluska, Gagliano & Witzany, 2018). For example, plants can overwrite the genetic code they inherited from their parents and revert to that of their grandparents or great-grandparents. This contradicts the traditional DNA-textbook conviction that offspring passively receive combinations of the genes carried by their parents. Under certain stress experiences, the plant can bypass unhealthy genetic sequences inherited from its parents and revert to the healthier sequences borne by its grandparents or great-grandparents ( Lolle *et al.,* 2005; Pearson, 2005; Weigl & Juergens, 2005).

With the rise of epigenetics, the context-dependent imprinting of experiences at both the phenotypic and the genotypic levels is an essential perspective to understand memory and learning in all organisms. Furthermore, memory and learning depend on a variety of successful communication processes within the whole organism ( Jablonka & Raz, 2009; Abraham *et al*., 2018; Patten *et al*., 2016).

Learning through memorized experiences to optimize behavior that can be inherited is well documented in all domains of life and does not fit the narrative of chance mutations (error replication) being key evolutionary drivers of genetic variations ( Rassoulzadegan & Cuzin, 2015; Spadafora, 2016). We now know that epigenetic switching outcompetes genetic mutations according to the requirements of the environmental context ( Gómez-Schiavon & Buchler, 2019; Stajic, Bank & Gordo, 2021). Transgenerational inheritance of such epigenetic memorized experiences opens the door to understanding the emergence of new capabilities which are not the result of replication errors (mutations) ( Liberman, Wang, & Greer, 2019; Braun *et al.*, 2020; Fitz-James & Cavalli, 2022). If we look at the roles of RNA networks in the evolution and development of organisms and epigenetic (re-)programming, a new concept appears more appropriate to integrate recent empirical data than the neo-Darwinist approach of the 20 ^th^ century ( Mattick, 2009, 2012; Shapiro, 2022).

Cellular organisms primarily try to survive by producing individual and social pathways embedded in situational contexts. This affects bodily impressions, actions and reactions which are relevant for epigenetic markings. At this stage, we have to realize that the transgenerational inheritance of epigenetically memorized and learned capabilities, in particular, does not fit the mutation/selection narrative of the modern synthesis, which is insufficiently complex to integrate that ( Witzany, 2021a).

## 5. Bridging the RNA world and cellular life: the virosphere

Last but not least, we have to identify how ancient RNA-world networks came into the cellular domains. Where do all these RNA regulators in cells come from? How did RNA get that crucial role for cell-based life ( Herbert & Rich, 1999, Herbert, 2004)? In this respect, it is important to identify those agents that implant the abundance of RNA stem-loops persistently in host genomes ( Villarreal, 2012a; Cech, 2012; Lehman, 2015; Atkins, Gesteland & Cech, 2010; Yarus, 2011).

### 5.1 The dominating planetary virosphere drives cellular evolution

After nearly a hundred years of cell biology, it is usual to think about life on this planet as cellular life forms that colonized nearly all ecological niches. All definitions of life have focused on cellular life. We know three domains of cellular life which assemble all kinds of cell-based life, from simple archaea to the most complex brain organs in humans. What appears to be still unusual is thinking about life as a virosphere dominating the whole planet, in so far as viruses outnumber cellular life forms tenfold ( Villarreal, 2005; Wolf & Koonin, 2007; Forterre & Prangishvili, 2009). In 1 milliliter of seawater we find 1 million bacteria but ten times more viruses. We must make it clear to ourselves that this planet is a sea of viruses with rare islands representing cellular life. Viruses are clearly more abundant and diverse than their targets, which means that the host cells relevant to the infection are a rare resource within an extremely competitive virosphere ( Koonin & Krupovic, 2018; Krupovic, Dolja & Koonin, 2020).

The early quasispecies, RNA viruses and related genetic parasites can be considered as key drivers of evolutionary processes in the evolution of all domains of life. Viruses have colonized all cellular organisms since the beginning of life ( Suttle, 2013; Rohwer & Barott, 2013). In most cases, this is a persistent lifestyle which does not harm the host. Persistent viruses remain as regulatory tools (exapted or coopted), and most of them remain as defectives, which means even fragmented they are effective agents in cellular DNA habitats such as the whole variety of mobile genetic elements and related genetic parasites ( Ryan, 2009; Forterre, 2010; Villarreal, 2009b; Roossinck, 2012). We speak about self-splicing introns and an abundance of non-coding RNAs such as SINEs, LINES, Alus, LTRs non-LTRs, retroposons and many others. They may further fragmentize and reassemble, insert and delete, combine and recombine nucleotide sequences without damaging the protein-coding genes which generate the development and growth of cellular phenotypes. They really are masters of natural genome editing in all its steps and substeps ( Lambowitz & Zimmerly, 2011; Villarreal & Witzany, 2018; Vignuzzi & Lopez, 2019; Benler & Koonin, 2022; Stedman, 2015; Witzany, 2009). In addition, the various forms of viruses from single-stranded RNA viruses up to double-stranded DNA viruses may combine and recombine their genomic features. Such skills are completely absent in cellular domains of life. Their rich social lives are also coordinated by communication processes ( Witzany, 2011b; Erez *et al*., 2017; Sanjuan, 2021). Persistent infection-derived non-coding RNAs are the main drivers of all single steps in epigenetic imprinting and execution of all related processes ( Conley & Jordan, 2012; Slotkin & Martienssen, 2007).

Meanwhile, we know many examples in which viruses and related genetic parasites are key drivers for the evolution of new features in organisms. Well-known examples are the evolution of the placenta of mammals with its retroviral-derived syncytin genes, or the arc proteins, retroviral-derived proteins essential for synaptic plasticity in animals ( Bonnaud *et al*., 2004; Villarreal, 2016a; Day & Shepherd, 2015; Hantak, Einstein, Kearns & Shepherd, 2021). Even the evolution of innate and adaptive immune systems depends crucially on persistent viral infections ( Villarreal, 2009c, 2011; Broecker & Moelling, 2019). Furthermore, the split between great apes and humans in their evolutionary history may have been initiated by waves of viral infections ( Villarreal, 2004; Witzany, 2021b). Many other examples of viral genome editing by invention and integration of new genes which are currently known include replicase, polymerase, integrase, DNA repair, restriction/modification, methylation, a bilayer nuclear envelope, division of transcription and translation, nuclear pores, tubulin-based chromosome duplication, chitin, calcification, linear chromosomes, cartilage and bones, skin, dermal glands for poison, mucus and milk, larvae, egg and flowering plants ( Villarreal, 2005; Atkins, Gesteland & Cech, 2010).

Lynn Margulis showed convincingly that the eukaryotic cell is not the result of a series of selected replication errors but a social compound of former free-living prokaryotes ( Margulis, 1992). A further question remains the origin of the eukaryotic nucleus, where several new features are present that are completely absent in prokarytic cells but that are present in a variety of DNA viruses ( Colson *et al.,* 2018) The precursor of the eukaryotic nucleus may derive from a large double-stranded DNA virus that persistently colonized a prokaryotic host ( Villarreal & DeFilippis, 2000; Chaikeeratisak *et al*., 2017; Takemura, 2020; Bell, 2020) The hosting cell must have lost its cell wall, with the virus incorporating the prokaryotic genes into its pre-nuclear genome, particularly in cases of encoding for metabolism and translation. This virus coordinated both its own replication and its transcription genes. (Interestingly, the α -proteobacteria-derived mitochondria resisted genetic integration and still replicate by themselves but in concert with the host cell.) Such a large double-stranded DNA virus presented a double-layered membrane and a tubulin system. Additionally, such a large DNA virus could integrate the conserved functions of the other unified prokaryotic partners of the eukaryotic cell into a coherent nucleotide line-up of a genetic identity which ensures well-coordinated interacting parts.

### 5.2 Persistent viral life style through addiction modules

A helpful model to understand the persistent lifestyle of viruses and related genetic parasites in cellular organisms is the “addiction module”. This presents an empirically based explanatory model which may be intrinsic to all regulations/counter-regulations in cellular organisms. Currently we can identify such addiction modules in toxin/antitoxin, restriction/modification and several insertion/deletion modules. They derived from infectious viral clouds and competing viral clouds that, together with the host immune system, reach an equilibrium status which is missing in the members of the species which did not experience these infection waves and their remnants. These addiction modules change the genetic identity of the host organism. Members of the same species that integrated such addiction modules into their genetic identity are protected against the toxin, whereas members without this integration are exposed to the toxin ( Villarreal, 2012b, 2016b; Lehnherr, Maguin, Jafri & Yarmolinsky, 1993; Engelberg-Kulka & Glaser, 1999; Kobayashi, 2001; Mruk & Kobayashi, 2014).

## 6. Self-organization of matter and self-empowerment of life

Together with the RNA remnants of former genetic parasites (defectives), such as long non-coding RNAs and the abundance of short non-coding RNAs or microRNAs and their dominant roles in gene regulation of all cellular processes such as replication, transcription, translation, repair, immunity, epigenetics and additionally the several substeps, we must acknowledge that, without RNA stem-loop group interactions, life as we know it on our planet would not function in detail and as a whole ( Mattick *et al*., 2023). The complementary multiple functions of RNA networks, viruses and cellular life empower life as a whole to adapt and evolve. This means that the early narrative of “self-organization of matter” should be adapted to “self-empowerment of life”. The step from abiotic physical reactions (“matter”) to biotic behavior (“life”) is not a gradual but a fundamental one, because in life we find basic interactional motifs that are really new and which are completely absent on abiotic planets. The new features do not represent gradual extensions or leaps in the quantity of former abiotic features but represent really new ones.

On looking at the processes where RNA stem-loop group interactions produce random variations, we now have to decide to further term them as a number of errors in replication processes that lead to selection-relevant results. We may now look at such variations as the results of high productivity of random variants. Such variants then become objects to selection processes out of an abundance of varieties of competing and cooperating RNA stem-loop groups. A prominent example are the ribosomal subunits with their different parts, all of which have different historical roots. The inherent high productivity of RNA group behavior then may change the narrative of errors and error thresholds into high productivity and excess productivity. Excess productivity is defined by a limit on the number of base-pairs which a self-replicating RNA stem-loop group may have, before productivity output will not lead to a beneficial increase of information but will destroy the represented information in the following generations of the RNA population. This means that RNA stem-loop groups are able to perform an endless, sometimes opposing and incomputable, number of random productivity results, which are relevant to selection processes and together build the basics for self-empowering life to adapt, reproduce and survive. But if the productivity is too high, self-empowerment decreases.

Although high productivity is not a directed process, via biological selection it empowers living agents to adapt and evolve into biological complexity and an abundance of species throughout all domains of life on the genotypic and phenotypic levels ( Briones, Stich & Manrubia, 2009; Mercer & Mattick, 2013).

The differentiation between the molecular biological entities of the 20 ^th^ century and the socially interacting agents (communication of RNA networks, cells and viruses) based on the 21 ^st^ century denotes a paradigmatic change in explaining and understanding life (see Table 1).

**Table 1.** Different paradigms explaining and understanding life (reworked from Witzany, 2019).

Concept of	Molecular biology	Biocommunication
“Dead”	Pre-biotic chemical reactions	No sign-mediated interactions
Origin of life	Self-organization of matter	Self-empowerment of life
“Living”	Replication/biological selection (molecular reactions)	Sign-mediated interactions (social events)
Determinants	Natural laws (thermodynamics)	Semiotic rules
RNA ensembles	Molecular assembly	Agent-groups integrate or preclude non-self agents
Viruses	Escaped selfish parasites	Essential agents of life
Genetic variation	Error replication	RNA interaction based high productivity
Genetic novelty	Random mutations	Viruses and subviral RNA-networks edit code
Biological selection	Fittest type	Fittest consortium
Genetic code	Genetic material	Semiotic text (according syntax, pragmatics, semantics)
Biological information	Shannon entropy; content depending on molecular syntax	Content depending on contextual use by competent agent-groups
Communication	Information transfer *via* coding/decoding mechanisms	Agent-based social interactions mediated by signs according semiotic rules
Definition of “life”	Machine-like statistical mechanics	Social event realized by communicative interactions

## 7. Conclusions

The mechanistic narrative of error replications (mutations) being key drivers of genetic/genomic novelty is insufficiently complex to integrate RNA productivity. This is shown by our current knowledge about the pre-cellular emergence of the RNA world, the dominant role of persistent viruses, their relatives and defectives, such as mobile genetic elements, and their descendants, such as the abundance of non-coding RNAs and their role in gene regulation, generation of variants and genetic novelty, together with knowledge of the variety of epigenetic programming and the transgenerational inheritance of acquired characteristics.

RNA networks, viruses and their defectives constantly produce new genetic variants and generate new sequences which are exposed to selective forces. This constant production is an innovative process, not an error in pre-existing DNA sequences, and therefore should be denoted more precisely as high productivity being the essential driver of random variations. What was described by Manfred Eigen as self-organization of matter, we can now modify and call the self-empowerment of life, because the competencies and behavioral motifs of RNA stem-loop groups empower all organisms which host such groups to evolve, develop, adapt and perform all the regulatory processes necessary for all known life processes. As this high productivity is not part of the modern synthesis of evolution, it is time to develop an integrative theory of evolution which has better explanatory power to integrate the abundance of empirical data on RNA biology, virology and biocommunication of cells produced in recent decades.

## Data Availability

All data underlying the results are available as part of the article and no additional source data are required.
