# Supplementary material for: Self-empowerment of life through RNA networks, cells and viruses
Source: F1000Res. 2023 Mar 6;12:138. Originally published 2023 Feb 6. [Version 2] doi: 10.12688/f1000research.130300.2 (PMC9918806; doi:10.12688/f1000research.130300.2)
Supplement: Peer review history

# Peer review: Reviewer response for version 1

**DOI:** 10.5256/f1000research.143047.r162512

**Published:** 2023-02-24

**Authors:** Jordi Gomez Castilla

**Affiliations:** 1 Instituto de Parasitología y Biomedicina ‘López-Neyra’ (CSIC), Granada, Spain

This author is in complete agreement and enthusiastic about the change of the narrative of life to a more vital (although not-vitalistic), non-mechanistic and non-only syntactic one. Nietzsche made the first advances in this line for biology taking man as if he were just another biological being where he soon encountered difficulties in thinking in only Darwinian terms.

 “Self-empowerment” was exactly the metaphysical alternative set by Nietzsche to confront Spinoza’s “self-preservation”, the concept that Nietzsche discovered to be the strong metaphysical support to Darwin's natural selection. Malthus' theory was rejected by Nietzsche because its simple to explain the whole life. I agree the “self-empowerment” concept can be proposed as a hypothesis for scientific research. And it can be formulated in scientific terms, which can be proposed to change M. Eigen's narrative of “self-organization of matter”, which has self-preservation in its focus. By accepting life as a dynamic of power relations, self-destruction is one of the possibilities. We - men - seem to be moving rapidly in this direction.

 The hypothesis is that what matters is no longer the individual who enters the struggle of life but winning in the struggle, for which individuals must sacrifice part of their own aspects. This agrees with the authors: from individual replicons, they will become "quasispeices" then “consortia of quasispeices", covalently-fixed consortia (tRNA), rRNA. Nietzsche, first observed that the struggle of life explained as a competition (a war of all against all) ignores the immense amount of co-operation in the struggle (simultaneous co-operation and competition) because this struggle is not to preserve one’s life, but rather in the main to achieve power:

*«My idea is that every specific body strives to become master over all space and to extend its force (its will to power) and to thrust back all that resists its extension. But it continually encounters similar efforts on the part of other bodies and ends by coming to an arrangement (‘union’) with those of them that are sufficiently related to it: thus they conspire together for power. And the process goes on»*

 “ *Darwin forgot the spirit (that is English!); ….. It will be noted that by "spirit" I mean care, patience, cunning, simulation, great self-control, and everything that is mimicry (the latter includes a great deal of so-called virtue*).”

 Communication is at the center for reaching agreements and social relations -“mimicry” is a communication element- and yes, at the center of self-empowering. Communication rules will change accordingly, the ‘organism’ interprets according to its biological interest in surviving and advancing itself. For this reason, Nietzsche’s perspectivism is not entirely relativist, but pragmatic.

 “There are no facts, only interpretations” /// “In fact, interpretation is itself a means of becoming master of something. (The organic process constantly presupposes interpretations.”

 Foucault and Canetti have also carried out the non-metaphysical formulation of power. In their definitions, Power is the action of a force on another force, and not the action of a force on an object. But, power as such does not exist, it does not accumulate, it is only present as a relation between two or more forces, and it manifests only in action. It normally refers to the government of behaviors.

 Minor points

 Page 4: “tripetide”, may be the authors wanted to say dipeptide.

 Page 7: “see chapter 3”  It is not clear what reference this chapter refers to.

 Page 9: “Such memory/learning skills can be found in organisms ranging from viruses…”  Add citation of Esteban Domingo. J Virol. 2000 Apr; 74(8): 3543–3547. Memory in Viral Quasispecies.

 Page 12: Table 1. “dead”. Probably better “inert”?

Is the review written in accessible language?

Yes

Are all factual statements correct and adequately supported by citations?

Yes

Are the conclusions drawn appropriate in the context of the current research literature?

Yes

Is the topic of the review discussed comprehensively in the context of the current literature?

Yes

Reviewer Expertise:

Biochemistry and Molecular Biology.

I confirm that I have read this submission and believe that I have an appropriate level of expertise to confirm that it is of an acceptable scientific standard.

# Response

**Published:** 2023-02-24

**Authors:** Guenther Witzany

**Affiliations:** Telos - Philosophische Praxis, Austria

Yes, Communication on all levels of sign-mediated interactions within and between RNA networks, viruses and cellular organisms together with epigenetic markings that ensure memory and learning empower biotic agents to survive beyond the outdated concept of mutations (error replications) and selection.  If better learned skills and competencies are relevant for survival, then it is clear that the old narrative is insufficiently complex to integrate that. An integrative theory of evolution has to outline this in future.

# Peer review: Reviewer response for version 1

**DOI:** 10.5256/f1000research.143047.r162511

**Published:** 2023-02-21

**Authors:** Ascensión Ariza-Mateos

**Affiliations:** 1 Department of Biochemistry and Molecular Pharmacology, New York University Grossman School of Medicine, New York, NY, USA

Villarreal and Witzany define a novel and thought-provoking concept in Biocommunications: Self-empowerment as the key feature during evolution and the core of biotic communication. They establish RNA stem-loop groups as essential molecules involved in the initial step between abiotic matter and life. These RNA stem-loops are the main characters of evolution, creating communicative interactions with themselves and other molecules by competition and cooperativity, building molecular consortia, and allowing the emergence of viruses, cells, and organisms. Life complexity is sustained by complex RNA networks, viruses, and molecular editing, constantly changing and adapting to each context, allowing biocommunication at all levels. As experts in Biocommunications, the authors argue for a more complex understanding of the role of RNA productivity, viruses, and epigenetics in shaping genetic variation and driving evolutionary change.

 The manuscript is thorough, and the authors show a deep and updated knowledge of RNA, Virology, and Evolution, besides providing a compelling and innovative perspective. From my point of view, the article is clear, but the authors assume all readers will have a wide knowledge of Biocommunications, and some ideas are based on concepts not sufficiently described in the text. I understand the difficulties of including all this information, but an introduction to the essential concepts may be helpful for a more fluent reading. Some examples are RNA syntax; “The Shooting of the Hunters”; “Umwelt”; Teleonomic key technique; Proponents; Turing, and von Neumann machines ideas; and “hypercycles”. Additionally, there is a concept that might be interesting to include in the paper regarding Biocommunication and context: Riboswitches. These are molecules found in all life realms that show conformational changes in response to a specific ligand, like a secondary metabolite, modulating transcription and translation.

 Overall, I consider this study of significant interest to the general readership of F1000Research journal.

 Finally, I would like to give a list of suggestions to improve the clarity and impact of the manuscript:

- Figure 1 provides ample information, but I have found some difficulties trying to understand it. Perhaps more details in the legend will improve its interpretation. Likewise, more details about the flow of arrows should be explained to clarify their meaning.
- I suggest changing the wording of RNAses to RNases. This is a common misuse, but the proper term is RNase.
- Section 2.1:
  - The text states that RNase H emerged from the RNA world and is introduced before the proteins’ emergence. It can be misunderstood as an RNA molecule, but this RNase is formed exclusively by protein.
  - The tri-peptide concept can be ambiguous and unclear for the reader. It would also be helpful to provide some additional explanation.
  - This section is especially engaging and covers a logical evolution in the interconnections between RNA activities and how they became more complex. However, everything is explained as if RNA was the only starter, whereas that occurs in collaboration with other types of molecules which appear during evolution, such as proteins, essential to make more complex relationships with RNA. Maybe the authors mean ribosome and RNase activities in RNA molecules, which evolve later in protein molecules.
- Section 2.4:
  - There is a typo, "startet" instead of started.
  - Please, consider a period after the reference (Witzany, 2015).
- Section 2.5: there is an absence of a period or comma after the references (Villarreal, 2009a; Villarreal & Witzany, 2021).
- Section 3:
  - I have found this section a little hard to understand, and I would appreciate some help from the authors to make it clearer. Especially the sentence: *The evolution of living systems with quasispecies dynamics and proteins depends on an unequivocal “code-system” and a relational protein system which evolves via “hypercycles”.*
  - There is a typo, "irreversibe" instead of irreversible (include the “l”).
- Section 4: Please, consider a period after (Frenkel-Pinter et al., 2022; Witzany, 2018).
- Section 5.1: I miss the presence of two periods in this section, after (Colson et al., 2018), and (Villarreal & DeFilippis, 2000; Chaikeeratisak et al., 2017; Takemura, 2020; Bell, 2020).

Is the review written in accessible language?

Yes

Are all factual statements correct and adequately supported by citations?

Yes

Are the conclusions drawn appropriate in the context of the current research literature?

Yes

Is the topic of the review discussed comprehensively in the context of the current literature?

Yes

Reviewer Expertise:

Structural Biology, RNA Structure, Virology, Hepatitis C Virus, RNA-protein Interactions, Translation Regulation, Fragile X syndrome.

I confirm that I have read this submission and believe that I have an appropriate level of expertise to confirm that it is of an acceptable scientific standard.

# Response

**Published:** 2023-02-23

**Authors:** Guenther Witzany

**Affiliations:** Telos - Philosophische Praxis, Austria

according the suggestions in the 2nd paragraph of this review:

 Important issues, but this would need another chapter to integrate all suggestions.

 further suggestion

 to Figure 1: will be integrated

 to RNase H: corrected

 to section 2.1: looks rather clear

 to section 2.4: corrected

 to section 2.5: corrected

 to section 3, 4 and 5.1: corrected

# Peer review: Reviewer response for version 1

**DOI:** 10.5256/f1000research.143047.r162510

**Published:** 2023-02-10

**Authors:** Felix Broecker

**Affiliations:** 1 Idorsia Pharmaceuticals Ltd., Allschwil, Switzerland

This article introduces the concept of "self-empowerment" of life, the key message of which is that life forms and their evolution cannot be explained if viewed as purely syntax (coding DNA/RNA) driven automatons (Turing or von Neumann machines). Instead, the specific context (pragmatics) of the syntax, which is governed primarily through RNA agents that communicate via stem-loop structures, is the more important aspect of life. RNAs regulate various life processes including epigenetics (and inheritance thereof), which represents an essential layer of context for the coding genes, which is subject to evolutionary selection and more important for evolutionary processes when compared to random mutations of coding genes. The article is well-written and conveys the message in a comprehensive yet concise manner. Overall the article is already in a very good shape. However, the authors may consider the following suggestions and comments:

 Page 3, around "An unexpected finding is that cooperative RNA stem-loops outcompete selfish ones". Looking at experiments and computational data published, for example, by Ichihashi ^1^^,^^2^ there is evidence that selfish or parasitic replicators (RNAs) do take over a population, leading to the collapse of the population, unless there is compartmentalization. Once compartmentalization is introduced, not only are the parasites prevented from eradicating the population, they are also actively contributing to the evolution of complexity. I suggest including this information and some relevant references by answering the question whether the models in which "cooperative RNA stem-loops outcompete selfish ones" also require compartmentalization.

 Page 4, bottom: Assuming that viruses and cells have common ancestors, that cells evolved from viruses or vice versa, or at least there is constant lateral gene exchange between virosphere and cellular life, do the authors have any explanation as to why there is not a single virus expressing a ribosome? Has the ribosome become too complex (optimized) in present-day cells, requiring dozens of accessory proteins? I am asking also in light of the finding that some of the NCLDVs were found to have genetic information (incomplete) of the translational machinery.

 Page 6, "The reverse transcriptase (…) seems to stem from a retroviral origin." In my understanding, retroviruses as we know them have relatively recently emerged evolutionarily. We know that a ribozyme-reverse transcriptase can evolve ^3^. RTs had existed probably long before the emergence of retroviruses.

 Page 7, "Empirical data contradict this model of explanation." – I suggest softening this to "There is no empirical data to explain this model." (or similar) unless there is actual contradicting data, in which case it should be briefly mentioned/explained.

 Page 7, around the paragraph starting with "Eigen’s adaptation…" Very interesting. Just a thought that came to my mind is that, for example, humans and mice share virtually the same set of genes (i.e., the syntax is so similar that you can easily "knock in" human genes into mouse cells and they will in most cases perform the exact same functions), yet the phenotype of both species differs substantially. The phenotypic differences are mostly the result of pragmatic context rather than amino acid sequence, i.e., syntax.

 Technical: There are some full stops missing throughout the manuscript, especially after citations.

Is the review written in accessible language?

Yes

Are all factual statements correct and adequately supported by citations?

Yes

Are the conclusions drawn appropriate in the context of the current research literature?

Yes

Is the topic of the review discussed comprehensively in the context of the current literature?

Yes

Reviewer Expertise:

Mobile genetic elements, (evolutionary) virology, vaccines, immunology

I confirm that I have read this submission and believe that I have an appropriate level of expertise to confirm that it is of an acceptable scientific standard.

## References

[1] : Plausible pathway for a host-parasite molecular replication network to increase its complexity through Darwinian evolution. *PLoS Comput Biol* .2022;18(12) : 10.1371/journal.pcbi.1010709 e1010709 10.1371/journal.pcbi.1010709 36454734PMC9714742

[2] : Darwinian evolution in a translation-coupled RNA replication system within a cell-like compartment. *Nat Commun* .2013;4: 10.1038/ncomms3494 2494 10.1038/ncomms3494 24088711

[3] : A reverse transcriptase ribozyme. *Elife* .2017;6: 10.7554/eLife.31153 10.7554/eLife.31153 28949294PMC5665644

# Response

**Published:** 2023-02-18

**Authors:** Guenther Witzany

**Affiliations:** Telos - Philosophische Praxis, Austria

ad page 3: Yes, we will include these arguments

 ad page 4: The issue of the ribosome and virus is indeed fundamental and interesting.  Although some viruses do enclose host ribosomes in virions and as noted NCLDV can encode many translational components, to date no virus appears to encode the ribosome itself.  Yet the ribosome itself clearly appears to be a collective of ligated stem-loop RNAs originally derived from various sources (in a virus-like process).  This implies that once the ribosome emerged, it became the fundamental host for all subsequent viruses as well as a fundamental entity for cells themselves.  Thus the clear and lasting virus-host dynamic emerged from the collective and transmissible RNA world and created a host that could acquire meaningful virus-derived code.

 ad page 6: Yes, important note! As Reverse Transcriptase based early RNA world Replicons predated the DNA world and the cellular world, and retroviruses emerged propably in a later stage of evolution there is no doubt, that retroviruses decended from these early reverse transcriptase sharing RNA replicons. We will note that (see: Koonin et al. Viruses Defined by the Position of the Virosphere within the Replicator Space)

 ad page 7: Empirical Evidence of the non-existence of von Neumann and Turing machines as well as theoretical considerations contradict this model of explanation. Nature of living organisms cannot be properly accommodated within such a theoretical framework. This is because the language that codes machine programs is not compatible with that of the genetic code. Languages controlling Turing and von Neumann machines are formal algorithms, in which syntax determines meaning independently of context. Gene expression depends on environmental context, however, so cannot be similarly treated as a formal language.

 ad Page 7, around the paragraph starting with "Eigen’s adaptation…": Yes, as most presentations at the meeting in Salzburg 2022 (How Evolution Learnt to Learn) demonstrated the phenotypic differences depend on epigenetic markings, which indeed are the result of pragmatic context of different lifeworlds (of e.g. humans and mice)

 ad Technical: Will be corrected

# Peer review: Reviewer response for version 1

**DOI:** 10.5256/f1000research.143047.r162513

**Published:** 2023-02-10

**Authors:** Frantisek Baluska

**Affiliations:** 1 Plant Cell Biology, University of Bonn, Bonn, Germany

This is very important and interesting manuscript illuminating diverse roles of RNA networks in evolution and development of organisms in all domains of life. Cooperative evolution of viruses, cells and organisms is under-appreciated but it allows understanding a the most complex issues related emergence and evolution of life. This RNA sociology places life in a correct context and explains many mysteries left behind from the outdated 29th century concepts based primarily on statistical mathematics. Life is not based on mechanistic machines or automatons but is based on truly living organism which actively experience and interpret their ever-changing and very complex abiotic and biotic environment. All organisms are based on cells which communicate, learn and memorize their experiences. I have only three small comments:

 1) Authors could briefly discuss the still confusing issue if viruses are living or non-living agents. Also, why viruses act as living agents only if they invade living cells and seems to be inert (non-living) outside of cells.

 2) For the alternative endosymbiotic origin of the eukaryotic nucleus see Baluška F, Lyons S (2021) ^1^.

 3) Roles of viral infections in the evolution of cells were discussed earlier. Baluška F (2009) ^2^.

Is the review written in accessible language?

Yes

Are all factual statements correct and adequately supported by citations?

Yes

Are the conclusions drawn appropriate in the context of the current research literature?

Yes

Is the topic of the review discussed comprehensively in the context of the current literature?

Yes

Reviewer Expertise:

Cell Biology, Plant Sensory Biology, Evolution

I confirm that I have read this submission and believe that I have an appropriate level of expertise to confirm that it is of an acceptable scientific standard.

## References

[1] : Archaeal Origins of Eukaryotic Cell and Nucleus. *Biosystems* .2021;203: 10.1016/j.biosystems.2021.104375 104375 10.1016/j.biosystems.2021.104375 33549602

[2] : Cell-cell channels, viruses, and evolution: via infection, parasitism, and symbiosis toward higher levels of biological complexity. *Ann N Y Acad Sci* .2009;1178: 10.1111/j.1749-6632.2009.04995.x 106-19 10.1111/j.1749-6632.2009.04995.x 19845631

# Response

**Published:** 2023-02-18

**Authors:** Guenther Witzany

**Affiliations:** Telos - Philosophische Praxis, Austria

ad 1: As viruses seem to predate cellular life and together with RNA networks invented cellular life and additionally outnumber cellular life 10 times and infect cells multiple throughout their lifes it would extend energy costs to still carry on cell-independent replication apparatus, which could have led to an evolutionary arms race between cell-dependent and cell-independent replicating viruses. Viruses invented the cellular life-sphere that guarantees their replication. The cellular biosphere on this planet is the dominant lifesphere of viruses. This is the benefit for current viral life styles. We have outlined this definition problem of viruses being alive or not in chapter 6 of: Villarreal LP, Witzany G. Social Networking of Quasi-Species Consortia drive Virolution via Persistence. AIMS Microbiol. 2021 Apr 30;7(2):138-162.

 ad 2+3: We will insert suggested references
